# *EPHA2* Segregates with Microphthalmia and Congenital Cataracts in Two Unrelated Families

**DOI:** 10.3390/ijms22042190

**Published:** 2021-02-22

**Authors:** Philippa Harding, Maria Toms, Elena Schiff, Nicholas Owen, Suzannah Bell, Ian Christopher Lloyd, Mariya Moosajee

**Affiliations:** 1 Institute of Ophthalmology, University College London, London EC1V 9EL, UK; philippa.harding.17@ucl.ac.uk (P.H.); maria.toms.14@ucl.ac.uk (M.T.); n.owen@ucl.ac.uk (N.O.); 2 The Francis Crick Institute, London NW1 1AT, UK; 3 Moorfields Eye Hospital NHS Foundation Trust, London EC1V 2PD, UK; elena.schiff@nhs.net (E.S.); suzannah.bell@nhs.net (S.B.); 4 Great Ormond Street Institute of Child Health, University College London, London WC1N 1EH, UK; IanChristopher.Lloyd@gosh.nhs.uk; 5 Manchester Academic Health Sciences Centre, University of Manchester, Manchester, M13 9PT, UK; 6 Great Ormond Street Hospital for Children NHS Foundation Trust, London WC1N 3JH, UK

**Keywords:** *EPHA2*, microphthalmia, cataracts, congenital, eye, development, whole genome sequencing (WGS), next-generation sequencing (NGS), genetics, zebrafish

## Abstract

EPHA2 is a transmembrane tyrosine kinase receptor that, when disrupted, causes congenital and age-related cataracts. Cat-Map reports 22 pathogenic *EPHA2* variants associated with congenital cataracts, variable microcornea, and lenticonus, but no previous association with microphthalmia (small, underdeveloped eye, ≥2 standard deviations below normal axial length). Microphthalmia arises from ocular maldevelopment with >90 monogenic causes, and can include a complex ocular phenotype. In this paper, we report two pathogenic *EPHA2* variants in unrelated families presenting with bilateral microphthalmia and congenital cataracts. Whole genome sequencing through the 100,000 Genomes Project and cataract-related targeted gene panel testing identified autosomal dominant heterozygous mutations segregating with the disease: (i) missense c.1751C>T, p.(Pro584Leu) and (ii) splice site c.2826-9G>A. To functionally validate pathogenicity, morpholino knockdown of *epha2a*/*epha2b* in zebrafish resulted in significantly reduced eye size ± cataract formation. Misexpression of N-cadherin and retained fibre cell nuclei were observed in the developing lens of the *epha2b* knockdown morphant fish by 3 days post-fertilisation, which indicated a putative mechanism for microphthalmia pathogenesis through disruption of cadherin-mediated adherens junctions, preventing lens maturation and the critical signals stimulating eye growth. This study demonstrates a novel association of *EPHA2* with microphthalmia, suggesting further analysis of pathogenic variants in unsolved microphthalmia cohorts may increase molecular diagnostic rates.

## 1. Introduction

*EPHA2* (OMIM:176946) encodes a 976 amino acid transmembrane tyrosine kinase receptor [1]. EPHA2 is part of the ephrin (Eph) family of receptors, which are known to be widely expressed during early embryogenesis, where they play a key role in the development of neuronal and vascular networks, as well as epithelial tissues [2]. The Eph family contains 16 receptors, grouped into EphA and EphB, and eight ephrin ligands [3]. Eph receptors and ephrin ligands are both membrane-bound, mediating cell-contact-dependent bidirectional signalling [2]. They regulate several cellular processes, including adhesion, migration, morphology, proliferation, differentiation, survival, and secretion [4]. The EPHA2 protein binds to ephrin type A ligands, and has an extracellular region containing the ligand-binding domain, a cysteine-rich domain and two fibronectin domains, a transmembrane segment, an intracellular region consisting of a juxtamembrane domain, a tyrosine kinase domain, a sterile alpha motif (SAM) domain and postsynaptic density protein, disks large, and a zona occludens (PDZ)-binding motif [2,5,6,7]. EPHA2 is important for the correct formation of multiple organs, and is highly expressed in the developing kidney, inner ear, and lens [3,6,8,9].

Lens fibre cells are highly organised and tightly packed to support lens transparency [10]. Their ordered structure is maintained through extensive cell–cell adhesion complexes, including adherens, tight, and gap junctions [10,11]. These intercellular junctions sustain transparency in the avascular lens by transporting ions, solutes, nutrients, and water between the cells, and removing waste products [3,10]. Cadherin activity in adherens junctions is key for lens vesicle formation, as conditional deletion of *N*- and *E*-*cadherin* from the presumptive lens of mice results in lens defects [12]. Mouse models show that the loss of EphA2 function disrupts the N-cadherin-dependent intercellular adherens junctions that regulate lens fiber cell-cell interactions, causing altered cell shape and irregular lens structure due to weakened cellular connections, resulting in progressive cataracts appearing from 3 months of age [2,5,13]. This demonstrates an important role for EPHA2 in lens homeostasis through regulating adherens junctions, both during embryological development and throughout life [2,3,5,7,10].

Cataracts are lens opacities resulting from disruption of normal lens protein structure and/or function [14]. Congenital cataracts arise during embryonic development or in early childhood, with a global prevalence of 1–15 per 10,000 children [15]. Cataracts have a wide differential diagnosis, including maternally derived infections, iatrogenic, and trauma. However, a molecular cause can be identified for the majority of patients in 1 of 115 associated genes [14,16,17,18]. Inherited cataracts most commonly occur on their own (70%), but patients may also present with associated ocular (15%) and systemic features (15%). Common additional ocular features include anterior segment dysgenesis, retinal dystrophies, aniridia, and microphthalmia (defined as a small underdeveloped eye, with an axial length of more than two standard deviations below the age adjusted mean) [18,19]. Microphthalmia with cataracts have been associated with mutations in *CRYAA*, *CRYBA4*, *CRYBB1*, *CRYBB2*, *CRYGC*, *CRYGD*, *GJA3*, *FOXE3*, *PITX3*, *IPO13*, *SIPA1L3*, *VSX2*, *GJA8*, and *NHS* [16,20,21,22,23,24,25,26,27].

Cat-Map (https://cat-map.wustl.edu/home/cat-map-variant-file/-August (accessed on 1 August 2020)) reports 22 variants of *EPHA2* associated with congenital cataracts (OMIM #116600) through autosomal dominant and recessive inheritance, in addition to sporadic mutations. These pathogenic variants are associated with additional ocular disorders, including microcornea, lenticonus, and persistent fetal vasculature. However, no variants of *EPHA2* have previously been reported in association with microphthalmia. Microphthalmia has a complex aetiology with over 90 associated monogenic causes, in addition to large chromosomal rearrangements in 10–20% of patients [28,29,30,31,32,33]. Environmental factors, such as congenital infection, prenatal vitamin A deficiency, or teratogen/alcohol exposure, are also known to cause microphthalmia, although they are thought to contribute to a minority of cases [34,35,36,37]. Despite this, only 20–30% of all microphthalmia/anophthalmia patients obtain a genetic diagnosis, indicating that further studies are required to identify the disease mechanism [29,30,34,38,39]. Expanding microphthalmia aetiology is vital to elucidating genotype–phenotype correlations, as well as providing patients with personalised clinical care and genetic counselling.

In this study, we identified through next-generation sequencing (NGS) autosomal dominant *EPHA2* variants segregating congenital cataracts and bilateral microphthalmia in two unrelated families: (i) missense variant c.1751C>T, p.(Pro584Leu) and (ii) splicing variant c.2826-9G>A. Using zebrafish, the pathogenic effect of *EPHA2* disruption on axial length was functionally validated, as knockdown of *epha2*a and *epha2b* using morpholino antisense oligonucleotide technology resulted in a small eye phenotype. We therefore demonstrate a novel causative association of *EPHA2* with microphthalmia.

## 2. Results

### 2.1. Clinical Phenotype

#### 2.1.1. Family 1

**Figure 1:**
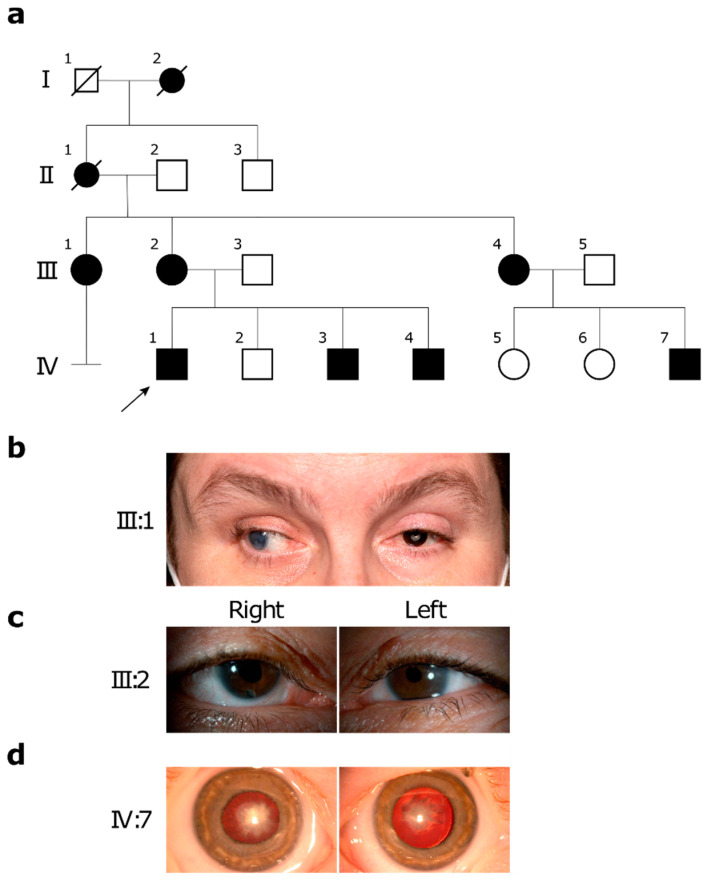
Pedigree and clinical images of family 1 identified as heterozygous for missense mutation *EPHA2* c.1751C>T, (p.Pro584Leu). (**a**) Pedigree of family 1 with bilateral microphthalmia and congenital cataracts. The proband is indicated with an arrow. Solid symbols indicate affected individuals and blank symbols indicate unaffected individuals. Women are represented by circles. Men are represented by squares. Deceased family members are indicated by a slash. (**b**) Clinical image of microphthalmia in patient III:1, age 47, following multiple needling and lens aspiration procedures in childhood, leaving her bilateral aphakic, followed by multiple ocular surgeries including endothelial keratoplasty and cyclodiode with right amblyopia, corneal decompensation, and secondary exotropia with hand movements vision, and a left keratoprosthesis and baerveldt tube with best corrected visual acuity (BCVA) of 0.9 LogMAR in the left eye. (**c**) Clinical image of microphthalmia in patient III:2, age 44, following similar needling and lens aspiration in childhood, followed by multiple ocular surgeries for aphakic glaucoma and right exotropia; the BCVA in the right eye was 1.10, and the left eye was 1.00 LogMAR. (**d**) Clinical image of bilateral irregular nuclear and cortical cataracts in patient IV:7 age 2.

The first family spanned four generations and consisted of nine affected individuals of white-British ethnicity (Figure 1a). The affected family members displayed non-syndromic bilateral mild microphthalmia, congenital cataracts, and nystagmus with an autosomal dominant inheritance pattern (Table 1, Figure 1). The proband patient IV:1 was sent for whole genome sequencing (WGS) along with both parents (III:2 and III:3) to complete the parent/offspring trio. Additionally, samples from patients III:1, IV:3, and IV:4 were sent for segregation. All affected individuals were found to be heterozygous for missense mutation c.1751C>T, (p.Pro584Leu) in *EPHA2*. All unaffected individuals were found to match the reference genome. No pathogenic variants were identified in any other known microphthalmia-associated loci, specifically *RAB3GAP2* and *FOXE3*, also located on chromosome 1. This family was previously included in a study evaluating real-world molecular diagnostic rates of developmental eye disorders, but a detailed description of their clinical phenotype and molecular diagnosis has not been published [40].

#### 2.1.2. Family 2

**Figure 2:**
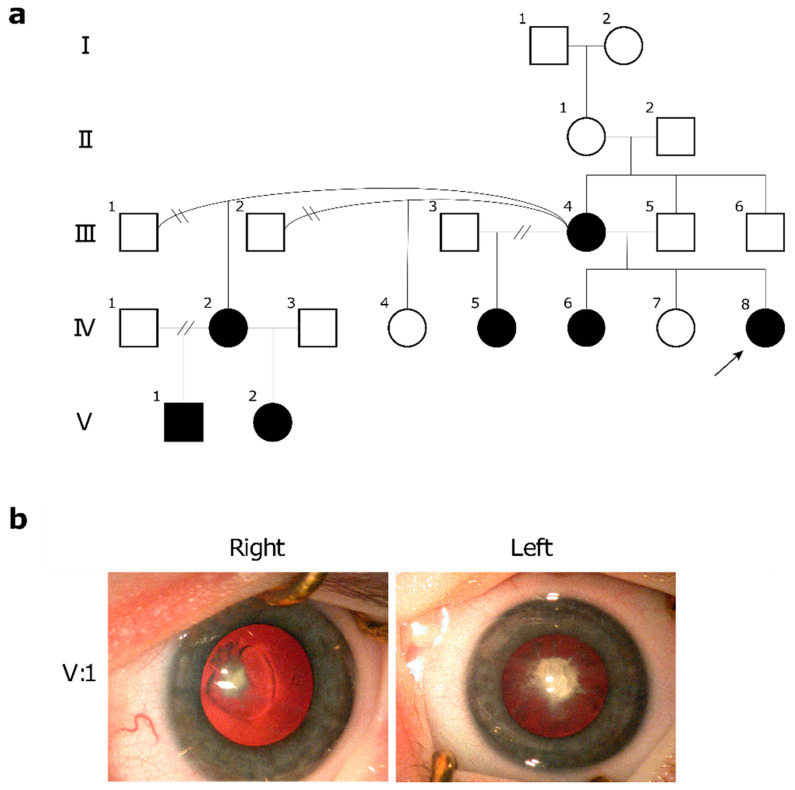
Pedigree and clinical images of family 2 identified as heterozygous for splicing variant *EPHA2* c.2826-9G>A. (**a**) Pedigree of family 2 with bilateral microphthalmia and congenital cataract. The proband is indicated with an arrow. Solid symbols indicate affected individuals and blank symbols indicate unaffected individuals. Women are represented by circles. Men are represented by squares. A break in the relationship line indicates the relationship no longer exists. (**b**) Clinical images of irregular nuclear and cortical lens opacities in patient V:1, age five, prior to removal.

The second family of white-British ethnicity incorporated six affected individuals across three generations (Figure 2a). Affected individuals exhibited non-syndromic bilateral mild microphthalmia and congenital cataracts, with an autosomal dominant inheritance pattern (Table 2, Figure 2). Four individuals exhibited nystagmus (IV:5, IV:6, IV:8, and V:1) and two patients suffered secondary aphakic glaucoma (IV:5 and IV:8). Patient IV:8 underwent the cataract-related targeted gene panel test, and was identified as heterozygous for splicing variant *EPHA2* c.2826-9G>A.

### 2.2. Knockdown of Zebrafish *EphA2* Genes Results in Microphthalmia

**Figure 3:**
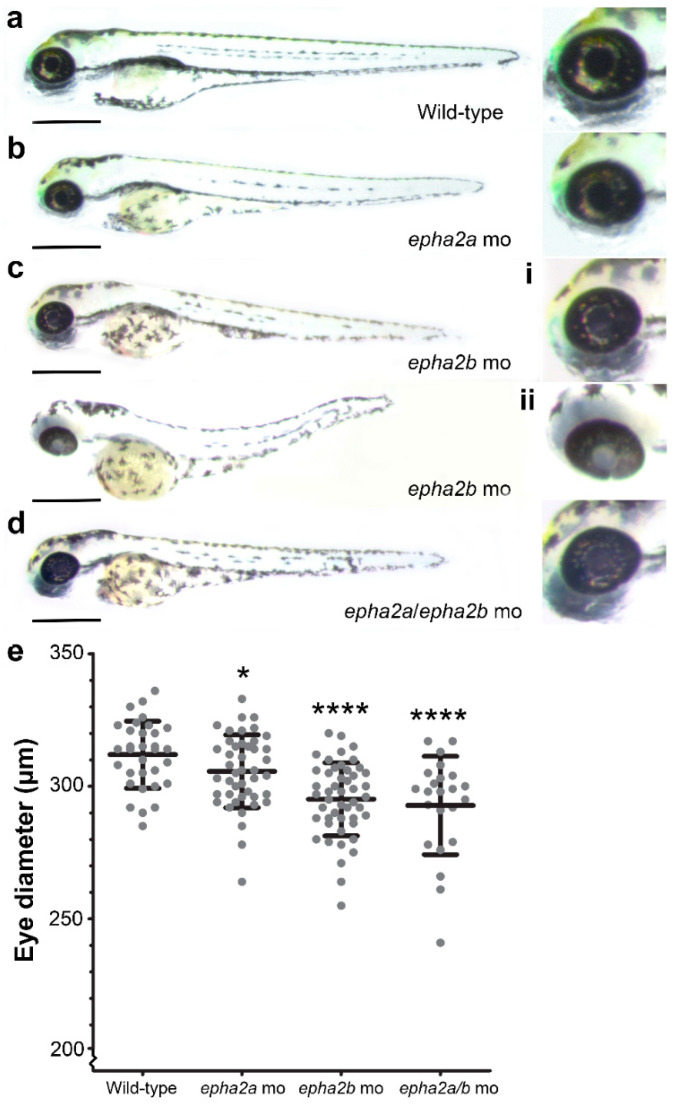
Reduced eye size in *epha2a* and *epha2b* morphant zebrafish. (**a**) Uninjected wild-type control embryos at 3 dpf; (**b**) morpholino knockdown of *epha2a*, (**c**) *epha2b,* and (**d**) *epha2a*/*epha2*b resulted in reduced eye size. Variable phenotype was seen with *epha2b* morphants, some with milder (i) or more severe eye (ii) features including lens opacification and coloboma. (**e**) Eye diameter measurement at 3 dpf showed a significant decrease in *epha2a*, *epha2b* and *epha2a*/*epha2b* morphants compared to uninjected wild-type controls. Unpaired t-tests were used to compare data. * *p* < 0.05, **** *p* < 0.0001. Scale bar = 500 µm.

To functionally demonstrate a pathogenic effect of *EPHA2* disruption on eye size using zebrafish, morpholino knockdown of the duplicated zebrafish orthologues, *epha2a* and *epha2b*, was performed. At 3 days post fertilisation (dpf), significantly reduced eye size was observed in the injected zebrafish (Figure 3); *epha2a* and *epha2b* morphant embryos had a mean eye diameter of 306 ± 13.7 µm (n = 44, *p* < 0.05) and 295 ± 13.8 µm (n = 48, *p* < 0.0001), respectively, compared to 312 ± 12.7 µm (n = 32) in age-matched wild-type siblings. The *epha2b* morphants were more severely affected, but there was phenotypic variability; lens opacification was noted in 44%, and 20.8% displayed severe microphthalmia with an eye diameter of <286.6 µm (which was >2 standard deviations below the mean of age-matched wild-type controls), with a persistent colobomatous defect in these embryos (Figure 3c). Double knockdown of *epha2a* and *epha2b* was also performed, resulting in a mean eye diameter of 292 ± 18.5 µm (n = 23, *p* < 0.0001) at 3 dpf. All zebrafish morphants showed a decrease in mean body length (Figure S1). To assess potential non-specific toxic effects of the morpholino injections, knockdown of p53 and *epha2b* was carried out simultaneously, causing reduced eye size consistent with knockdown of *epha2b* alone (Figure S2).

**Figure 4:**
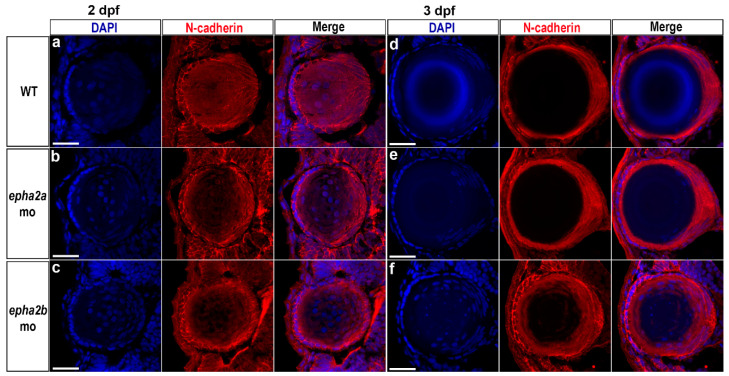
Lens abnormalities in *epha2b* morphant zebrafish. To assess lens development, N-cadherin (red) and DAPI (blue) staining were performed on lens sections from wild-type (WT), *epha2a*, and *epha2b* morphant zebrafish at 2 dpf (**a–c**) and 3 dpf (**d–f**); this permitted visualisation of the lens fibre cells and cell nuclei, respectively. At 2 dpf, there were no apparent differences between the morphants and uninjected wild-type siblings. At 3 dpf, the *epha2b* morphant showed retention of fibre cell nuclei within the lens (**f**), which was not observed in age-matched wild-type (**d**) and *epha2a* morphant larvae. Scale bars = 25 µm.

To investigate the lens abnormalities observed in *epha2b* morphants, N-cadherin and DAPI staining was performed to examine lens fibre cell localisation (Figure 4). At 2 dpf, *epha2a* and *epha2b* morphant lenses looked similar to age-matched wild-type siblings (Figure 4a-c). At 3 dpf, the e*pha2b* morphants showed retention of fibre cell nuclei within the lens (Figure 4f), while normal denucleation was observed in wild-type and *epha2a* morphant zebrafish at the same timepoint (Figure 4d,e).

## 3. Discussion

Here, we describe two unrelated families with autosomal dominant pathogenic variants in *EPHA2*, which segregate with bilateral congenital cataracts and microphthalmia: a four-generation family with missense mutation c.1751C>T, p.(Pro584Leu) identified using whole genome sequencing (WGS) (Figure 1, Table 1), and a two-generation family with splice site variant c.2826-9G>A identified through panel-based next-generation sequencing (NGS) (Figure 2, Table 2). Both of these variants are known to disrupt EPHA2 function and have been previously reported to cause isolated congenital cataracts [4,5]. However, to the best of our knowledge, no mutations in *EPHA2* have previously been associated with microphthalmia.

EPHA2 is a transmembrane tyrosine kinase receptor that mediates intercellular cadherin-based adherens junctions in the developing and mature lens [3,5,10,13]. The c.1751C>T, p.(Pro584Leu) variant was first described by Dave et al. in 2013 in a family with congenital bilateral nuclear lens opacities [5]. This mutation alters a highly conserved amino acid in exon 10, affecting the juxtamembrane region of the protein, in which autophosphorylation of tyrosine residues regulates the signalling activity of the receptor [5,6,41]. Splice site variant c.2826-9G>A was identified by Zhang et al. in a family with autosomal dominant congenital cataracts [4]. This single base substitution introduces a splice acceptor site, causing an intronic sequence of 7 bp to be included in the processed transcript, producing a polypeptide with an additional 71 amino acid residues at the C-terminal [4]. Further analysis demonstrated that this alternative protein shows stronger interaction with low-molecular-weight protein tyrosine phosphatase (LMW-PTP), a negative regulator of EPHA2 signalling, resulting in a loss of function.

Reciprocal signalling between the developing lens and optic cup during oculogenesis means pathogenic variants, which perturb lens development, may also disrupt the development of the ocular globe, resulting in complex ocular phenotypes, including comorbidity of cataracts and microphthalmia [28,30,42,43,44,45]. Other genes involved in forming intercellular junctions in the lens have pathogenic variants associated with both congenital cataracts and microphthalmia, for example, lens epithelial tight junction regulator *NHS* (OMIM:300457) and lens fibre gap junction component *GJA8* (OMIM:600897). Mutations in *NHS* can also cause Nance-Horan syndrome (OMIM:302350), with congenital cataracts, microphthalmia, dental anomalies, and intellectual disability [11,24,46]. Pathogenic *GJA8* variants cause Cataract 1, multiple types (OMIM:116200), including congenital cataracts and microphthalmia in both humans and mouse models when disrupted [21,47,48].

The pathogenic effect of *EphA2* disruption on eye size was functionally validated using morpholino-induced knockdown of *epha2a* and *epha2b* in zebrafish. At 3 dpf, morphant zebrafish were shown to have significantly reduced eye size compared to age-matched controls (*p* < 0.05) along with variable appearance of cataracts, suggesting that loss of *epha2* function plays a role in microphthalmia pathogenesis. The reduction in eye size in the majority of *epha2a/epha2b* morphants compared to age-matched controls was significant but not severe, reflecting the mild phenotype observed in both families. These results demonstrated a conserved role for EPHA2 in ocular globe and lens development, with disruption of *EPHA2* resulting in a small eye phenotype in both patients and animal models.

Previous work indicated that EPHA2 recruits actin to hexagonal cadherin/actin complexes in lens epithelial and fibre cells, which aid in aligning and packaging the cells to maintain a transparent structure. This organised structure is lost in knockout *EphA2*(-/-) mice, which display altered N-cadherin adhesion junctions and disruption of the actin cytoskeleton, leading to lens structure defects and cataracts [13,49]. Conditional deletion of *E*- or *N*-*cadherin* in the developing mouse lens leads to significantly smaller lens with epithelial and fibre cell defects, alongside a complex ocular phenotype including microphthalmia and iris hyperplasia [12].

In the present study, N-cadherin and cell nuclei staining at 3 dpf highlighted lens fibre cell defects in *epha2b* morphant zebrafish similar to that observed in other zebrafish cataract models, suggesting a conserved role in lens fibre cell development [50,51]. Mutations in *epha2* may disrupt the adherens junctions in the developing lens, which interfere with the molecular signalling of the optic cup, resulting in microphthalmia. However, the lens fibre cell abnormalities observed in the *epha2b* morphant larvae may be related to developmental delay caused by morpholino knockdown, and characterisation of *epha2b* germline mutants would be necessary to examine this further.

## 4. Conclusions

In this study, we identified functional variants of *EPHA2* in two unrelated families with bilateral mild microphthalmia and cataracts, and demonstrated a significant reduction in eye size of *epha2a/b* morphant zebrafish at 3 dpf. These findings reveal a novel association of *EPHA2* with microphthalmia, manifesting alongside cataracts, which is conserved across vertebrates. However, *EPHA2* is not typically found on microphthalmia, anophthalmia and coloboma (MAC) targeted gene panels, although it is included in cataract and lens-associated gene panels [52]; hence, patients undergoing genetic testing may remain unsolved. By including *EPHA2* in routine genetic testing of microphthalmia, a greater proportion of patients may receive a molecular diagnosis, which can inform genetic counselling and guide clinical management, ultimately providing patients with enhanced clinical care. The addition of *EPHA2* to the microphthalmia, anophthalmia and coloboma (MAC)-targeted gene panels may aid diagnostics, and should be considered for screening unsolved microphthalmic cases. Furthermore, these findings provide insights into the molecular pathways underlying microphthalmia, which will aid our understanding of disease pathogenesis and identification of potential targets for therapeutic development [28].

## 5. Materials and Methods

### 5.1. Ethical Considerations

This study had relevant local and national research ethics committee approvals (Moorfields Eye Hospital NHS Foundation Trust (MEH) and the Northwest London Research Ethics Committee), and adhered to the tenets of the Declaration of Helsinki. Patients and relatives gave written informed consent for genetic testing, through either the Genetic Study of Inherited Eye Disease (REC reference 12/LO/0141, 10/10/2016), or Genomics England 100,000 Genomes project (REC reference 14/EE/1112, 20/02/2015).

### 5.2. Mutation Identification

Patients underwent molecular testing performed in the clinical and research setting, using targeted gene panel testing (Cataract targeted gene panel of the Oculome; http://www.labs.gosh.nhs.uk/media/764794/oculome_v8.pdf (accessed on 22 February 2021) [52]) through the Rare & Inherited Disease Genomic Laboratory at Great Ormond Street Hospital (London, U.K.) and whole genome sequencing (WGS) as part of the U.K. Genomics England 100,000 Genomes Project. WGS datasets were generated using Illumina X10 sequencing chemistry. Aligned reads were utilised in calling SNV and small indels, as well as structural variants. Filtered annotated variants were prioritised in line with the ACMG (American College of Medical Genetics and Genomics ) and ACGS (Association for Clinical Genomic Science) best practices 2019. The sequence of *EPHA2* in the probands was compared to the reference sequence (GenBank accession NM_004431.5), and potentially disease-causing variants were assessed for segregation with the disease in Sanger-sequenced affected and unaffected family members. The results were reviewed by a multidisciplinary team (including molecular biologists, clinical geneticists, as well as the ophthalmology specialist managing the family), to confirm variant pathogenicity, prevalence in publicly available genome databases, and the clinical phenotype and mode of inheritance, before the molecular diagnosis was established.

On analysis, it was confirmed that no other microphthalmic patients in this database were found with mutations in *EPHA2*. In addition, other genes in the vicinity of *EPHA2* that were also in the MAC panels were analysed for any variants that may indicate a regulatory role or a cis/trans-acting modifying role.

### 5.3. Zebrafish Husbandry

Zebrafish (wild type) were bred and maintained according to local UCL (University College London) and U.K. Home Office regulations for the care and use of laboratory animals under the Animals Scientific Procedures Act at the UCL Bloomsbury campus zebrafish facility. UCL The Animal Welfare and Ethical Review Body approved all procedures for experimental protocols, in addition to the U.K. Home Office (License no. PPL PC916FDE7). All approved standard protocols followed the guidelines of the ARVO Statement for the Use of Animals in Ophthalmic and Vision Research Ethics [53,54].

### 5.4. Morpholino Design and Injection of Zebrafish

*epha2a* and *epha2b* splicing-blocking morpholino oligos previously used by Miura et al. were supplied by Gene Tools (Philomath, OR, USA) [55]. The sequences were as follows:

*epha2a*, 5’-GCAGTACATCTGAGAATCATATAAT-3’

*epha2b*, 5’-CAAAACCTTTTCACTTGCATTTACC-3’

2 pmol of *epha2a* or *eph2ab* morpholino, or 1 pmol of both, was injected into the yolk of one-cell-stage embryos. For p53 and *epha2b* co-knockdown, 0.25 pmol of p53 morpholino was injected per embryo.

### 5.5. Eye Measurement and Analysis

Horizontal eye diameter and body length were determined by imaging lateral views of anaesthetised larvae at 3 days post fertilisation (dpf). Images were analysed using ImageJ. For each group, the mean ± standard deviation was calculated. Wild-type and morphant data were compared using unpaired t-tests or Mann–Whitney tests; *p* < 0.05 was considered to be statistically significant.

### 5.6. N-Cadherin Staining of the Zebrafish Lens

Whole zebrafish larvae were fixed at 2 and 3 dpf in 4% PFA/PBS overnight at 4 °C before incubation in 30% sucrose/PBS overnight at 4 °C. The samples were mounted and frozen in TissueTek O.C.T (VWR International, Radnor, PA, USA) using dry ice. We cut 12 μm sections and collected them onto Superfrost PLUS slides (Thermo Fisher Scientific, Waltham, MA, USA). After air-drying for two hours, sections were washed in PBS/0.5% Triton-X before being blocked for 1 h with 20% normal goat serum (Sigma-Aldrich, St Louis, MO USA) in PBS/0.5% Triton-X and incubated in anti-N-cadherin antibody (Abcam #ab211126, Cambridge, UK) diluted 1:100 in antibody solution (2% normal goat serum in PBS/0.5% Triton-X) at 4 °C overnight. After washing with PBS/0.5% Triton-X, the sections were incubated in Alexa Fluor 568 secondary antibody (Abcam, Cambridge, UK) diluted 1:500 in antibody solution for 2 h at room temperature. Finally, the sections were washed, counterstained, and mounted using Prolong Diamond Antifade mountant + DAPI (Thermo Fisher Scientific, Waltham, MA, USA). The slides were imaged using a Leica LSM 710 confocal microscope.

## Figures and Tables

**Table 1:** Clinical phenotype data of four-generation family 1 with *EPHA2* mutation c.1751C>T (p.Pro584Leu) identified through whole genome sequencing segregating with bilateral microphthalmia and congenital cataract phenotype.

Patient	Age	Mutation	Clinical Phenotype								
			Microphthalmia (Eye Affected)	Axial Length (mm)		Visual Acuity (Most Recent)		Congenital Cataract	Other Ocular Findings	Surgery	
				L	R	L	R			Yes (Y)/No (N)	Complications
**I:2**	Dec.	Not sequenced	Bilateral	Unk.	Unk.	Unk.	Unk.	No	No	Unk.	Unk.
**II:1**	Dec.	Not sequenced	Bilateral	Unk.	Unk.	Unk.	Unk.	Yes	Nystagmus	Unk.	Unk.
**III:1**	47	c.1751C>T (p.Pro584Leu)	Bilateral	18.1	18.5	CF	0.78	Yes	Nystagmus Strabismus Epiretinal membrane	Y (bilateral lensectomies and multiple to manage ongoing complications)	Glaucoma following cataract surgery Corneal oedema/ decompensation Retinal detachment
**III:2**	44	c.1751C>T (p.Pro584Leu)	Bilateral	18.0	19.5	1.0	0.78	Yes	Nystagmus Strabismus	Y (bilateral lensectomies and strabismus surgery)	Glaucoma following cataract surgery
**III:3**	Unk.	No mutation	No	Unk.	Unk.	Unk.	Unk.	No	None	Unk.	Unk.
**III:4**	35	Not sequenced	Bilateral	Unk.	Unk.	Unk.	Unk.	Yes	Nystagmus Strabismus	Unk.	Unk.
**IV:1**	14	c.1751C>T (p.Pro584Leu)	Bilateral	Unk.	19.4	0.39	1.4	Yes	Nystagmus	Y (bilateral lensectomies)	None
**IV:2**	12	Not sequenced	No	Unk.	Unk.	Unk.	Unk.	None	None	Unk.	Unk.
**IV:3**	8	c.1751C>T (p.Pro584Leu)	Bilateral	20.80	22.87	0.39	0.39	Yes	Nystagmus	Y (bilateral lensectomies)	None
**IV:4**	4	c.1751C>T (p.Pro584Leu)	Bilateral	18.97	19.54	0.6	0.69	Yes	Nystagmus	Y (bilateral lensectomies)	None
**IV:7**	2	Not sequenced	Bilateral	15.15	15.05	Unk.	Unk.	Yes	Nystagmus, microcornea, aphakia	Y (bilateral lensectomies)	None

L; Left, R; Right, Dec.; Deceased, CF; Counting fingers, Unk.; Unknown.

**Table 2:** Clinical phenotype data of two-generation family 2 with *EPHA2* mutation c.2826-9G>A identified through next-generation segregating through NHS ocular malformation targeted (EYEMALF) gene panel with bilateral microphthalmia and congenital cataract phenotype.

Patient	Age	Mutation	Clinical Phenotype								
			Microphthalmia (Eye Affected)	Axial Length (mm)		Visual Acuity (Most Recent)		Congenital Cataract	Other Ocular Findings	Surgery	
				L	R	L	R			Yes (Y)/No (N)	Complications
**III:4**	Unk.	Not sequenced	Unk.	Unk.	Unk.	Unk.	Unk.	Unk.	Unk.	Unk.	Unk.
**IV:2**	8	Not sequenced	Bilateral	16.9	16.2	0.36	0.32	Yes	N	Unk.	Unk.
**IV:5**	16	Not sequenced	Unk.	Unk.	Unk.	0.66	0.48	Yes	Nystagmus, esotropia, post-operative glaucoma with optic disc cupping 0.1 bilaterally	Yes	Glaucoma
**IV:6**	22	Not sequenced	Unk.	Unk.	Unk.	0.6	0.48	Yes	Nystagmus, esotropia	Unk.	Unk.
**IV:8**	2	c.2826-9G>A	Bilateral	16.9	16.9	1.3	1.3	Yes	Nystagmus, microcornea, LE exotropia, large posterior lenticonus defects	Y (bilateral lensectomies)	Glaucoma with pupillary block
**V:1**	5	Not sequenced	Unk.	Unk.	Unk.	0.55	0.6	Yes	Nystagmus, esotropia	Y	None
**V:2**	2	Not sequenced	Bilateral	16.81	16.58	1.2	1.2	Yes	Steep corneas	Y	None

L; Left, R; Right, Unk; Unknown.

## Data Availability

The data presented in this study are available on request from the corresponding author.

## Supplementary Materials

The following are available online at https://www.mdpi.com/1422-0067/22/4/2190/s1.
