# Supplementary material for: EPHA2 Segregates with Microphthalmia and Congenital Cataracts in Two Unrelated Families
Source: Int J Mol Sci. 2021 Feb 22;22(4):2190. doi: 10.3390/ijms22042190 (PMC7926380; doi:10.3390/ijms22042190)
Supplement: Supplementary file 1 [file ijms-22-02190-s001.pdf]

Supplementary Materials:

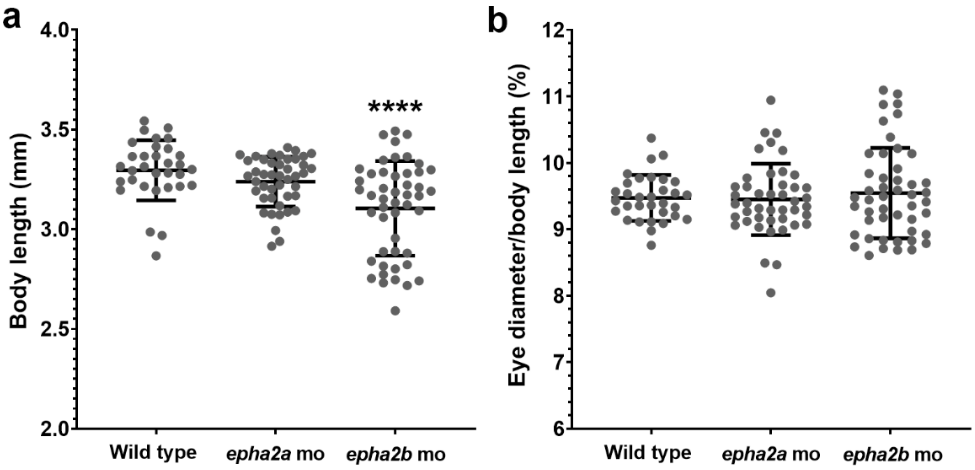

Figure S1. Body length in *epha2a* and *epha2b* morphant zebrafish. (**a**) At 3dpf, body length was found to be significantly reduced in *epha2b* morphant embryos ($p > 0.0001$) compared to un-injected controls siblings. The reduction in body length in *epha2a* morphants was not quite statistically significant ($p = 0.058$). (**b**) The eye diameter/body length ratio (%) was not found to be significantly different in the morphant groups compared to age-matched wild-type. Unpaired t-tests or Mann-Whitney tests were used to compare the data.

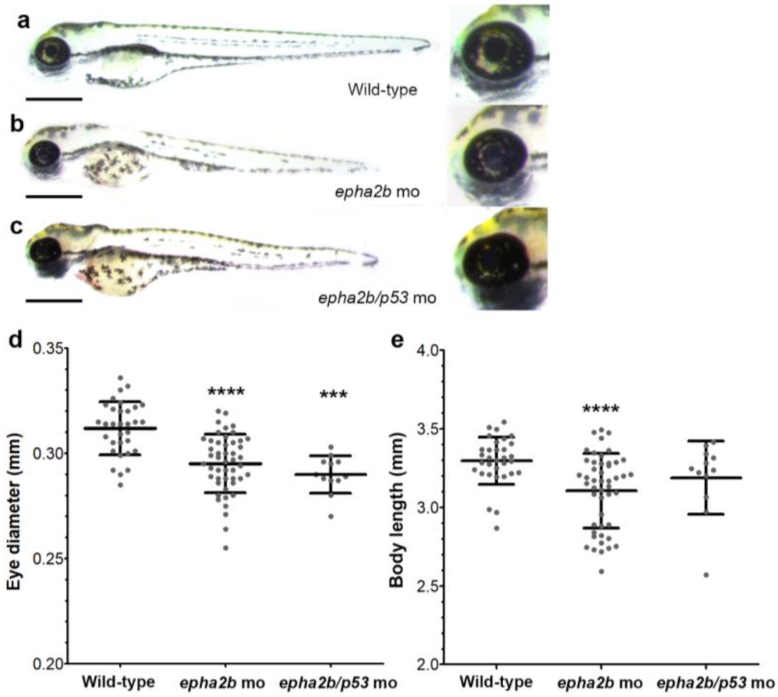

Figure S2. Knockdown of *epha2b* and *p53* in zebrafish embryos. (**a**–**c**) at 3dpf, simultaneous morpholino knockdown of *epha2b* and *p53* caused a similar microphthalmia phenotype to that observed in embryos with *epha2b* knocked down alone. (**d**) Eye diameter in *epha2b*/*p53* morphants was significantly reduced compared to uninjected wild-type and was not significantly different compared to *epha2b* morphants. (**e**) Body length was not significantly different compared to uninjected wild-type. **** $p < 0.0001$, *** $p < 0.001$.
